# Editorial: Computational pathology for precision diagnosis, treatment, and prognosis of cancer

**DOI:** 10.3389/fmed.2023.1209666

**Published:** 2023-06-06

**Authors:** Jun Cheng, Kun Huang, Jun Xu

**Affiliations:** ^1^National-Regional Key Technology Engineering Laboratory for Medical Ultrasound, Guangdong Key Laboratory for Biomedical Measurements and Ultrasound Imaging, School of Biomedical Engineering, Shenzhen University Medical School, Shenzhen University, Shenzhen, China; ^2^Medical Ultrasound Image Computing (MUSIC) Laboratory, Shenzhen University, Shenzhen, China; ^3^Marshall Laboratory of Biomedical Engineering, Shenzhen University, Shenzhen, China; ^4^Department of Biostatistics and Health Data Science, Indiana University School of Medicine, Indianapolis, IN, United States; ^5^Regenstrief Institute, Indianapolis, IN, United States; ^6^Institute for AI in Medicine, School of Artificial Intelligence, Nanjing University of Information Science and Technology, Nanjing, China

**Keywords:** computational pathology, digital pathology, machine learning, deep learning, medical image analysis, biomedical data analysis

Histopathology is considered the gold standard in determining the presence and nature of tumors. Technological advances in automated high-speed and high-resolution whole-slide imaging have laid the foundation for a digital revolution in microscopy. Digital histopathological images can be analyzed efficiently with image analysis and machine learning techniques. These techniques have shown great potential for extracting sub-visual, quantitative, and valuable features from whole-slide images to characterize tumors and support clinical decision (1, 2). Besides histopathological images, other data modalities, such as radiological images and multi-omics data, are also used to assist the decision-making process for cancer diagnosis, treatment, and prognosis (3, 4). At present, it is not clear how these macroscopic, microscopic, and molecular features are related. Exploring the association between different data modalities can give new insights into diseases.

This Research Topic is to highlight some latest developments in computational pathology that use either classical image analysis or state-of-the-art deep learning solutions for improved clinical decision making. A brief summary of the articles in this Research Topic is provided below.

Segmentation of regions of interest is usually an important step in the workflow of computer-aided diagnosis. Shi et al. collected a new enteroscope biopsy histopathological image dataset for image segmentation tasks and submitted it to a public data repository. This dataset contains 2,228 colorectal tissue images and their corresponding ground-truth annotations with the size of 224 × 224 pixels. To cover the transition process from normal to cancerous tissue, this dataset includes six tumor differentiation stages: normal, polyp, low-grade intraepithelial neoplasia, high-grade intraepithelial neoplasia, serrated adenoma, and adenocarcinoma. In this work, they compared the segmentation performance of five classical machine learning methods and three deep learning methods. Generally, the deep learning methods outperformed the classical machine learning methods by a large margin in all six tissue types. This study and the released dataset can serve as a good benchmark for colorectal histopathological image segmentation. Zhao et al. proposed a deep segmentation network to distinguish cancerous and intestinal metaplasia regions from normal gastric tissue. The segmentation results of multiple whole slide images from a specimen were mapped to the macroscopic image of the specimen. For a convenient use, they developed a software to automate the construction of mucosal recovery maps, which can expedite the learning process of early gastric cancer diagnosis.

Annotating pathological images requires professional knowledge and is time-consuming and costly. The annotations of most existing public datasets focus on the ground truth labels about what the diseases and lesions are, rather than why and how they are discovered and decided. Therefore, these datasets are not directly applicable for clinical use. To address this issue, Zhang et al. proposed a new annotation form, PathNarrative, which includes a hierarchical decision-to-reason data structure, a narrative annotation process, and a multimodal interactive annotation tool. PathNarrative can help collect both decision-to-reason labels and multimodal information on vision, language, voice, and behavioral trajectories. To verify the efficacy of this new annotation tool for human-AI collaborative diagnosis, they experimented on a colorectal pathological dataset with classification and captioning tasks. The experimental results show that the classification and captioning tasks achieve better performance with refined annotations, provide explainable details for doctors to make clinical decisions, and thus enhance doctors' trustworthiness and confidence to collaborate with artificial intelligence models.

Hu et al. performed a comparative study of gastric pathological image classification. They used a publicly available dataset, GasHisSDB, which contains three sub-datasets with different image sizes (80 × 80, 120 × 120, and 160 × 160 pixels). Seven classical machine learning classifiers and four deep learning classifiers were tested. For the classical machine learning classifiers, five feature extraction methods were used, including color histogram, luminance histogram, histogram of oriented gradient, local binary patterns, and gray-level co-occurrence matrix. Overall, the deep learning classifiers achieved much higher accuracy than the classical machine learning classifiers, no matter what kinds of features were used. In addition, they found that the deep learning classifiers misclassified different samples, implying that it is possible to use ensemble learning to obtain better predictive performance. Fully supervised methods require a sufficient quantity of images with annotations. However, in medical field it is difficult to collect and label data, which needs to be performed by experts. Wang et al. proposed a self-supervised learning method to classify malignant and non-malignant pathological images in eyelid melanoma. This method took advantage of a relatively abundant quantity of unlabeled data and a limited quantity of labeled data to learn features. In the self-supervised setting with a subset of images labeled, the proposed method achieved the best performance compared with five fully supervised methods.

Another popular research interest in computational pathology is to predict cancer survival based on quantitative image features and associate these features with molecular data. In a study by Couetil et al., interpretable histopathological features were extracted from whole slide images to predict 5-years survival and 5-years metastasis of melanoma. They used the morphological feature set described in a previous study (2) and introduced additional features to describe lymphocytes and other small, hyperchromatic cells. In total, 135 morphological features were extracted. Four classical machine learning models were implemented, including random forest, support vector machine, k-nearest neighbors, and logistic regression. This approach yielded a maximum F1 score of 0.72 and 0.73 for predicting survival and metastasis, respectively. Tumor-stroma reaction (TSR) is a critical feature in many solid tumors. Jiang et al. trained a serial of deep learning models to identify tumor vs. stroma regions and predict three types of TSR scores (fibrosis, stromal cellularity, and orientation of stromal cells) in ovarian carcinoma. Within the tumor-stroma interface region, they found that the TSR fibrosis scores were strongly associated with patient survival. Correlating the TRS fibrosis scores with gene expression data, they further found that the positively correlated genes were enriched in 14 KEGG pathways that are mostly associated with cancer signaling aberrations. This genotype-phenotype association analysis enables discovering the molecular basis of tissue morphological changes.

## Author contributions

Editorial writing: JC. Review and editing: JC, KH, and JX. All authors contributed to the article and approved the submitted version.
